# Carboplatin and oxaliplatin in sequenced combination with bortezomib in ovarian tumour models

**DOI:** 10.1186/1757-2215-6-78

**Published:** 2013-11-09

**Authors:** Zaynab Al-Eisawi, Philip Beale, Charles Chan, Jun Q Yu, Fazlul Huq

**Affiliations:** 1 Cumberland Campus, The University of Sydney, Lidcombe, Sydney, NSW 2141, Australia; 2 Sydney Cancer Centre, Concord Hospital, Sydney, NSW 2139, Australia; 3 Anatomical Pathology Department, Concord Hospital, Sydney, NSW 2139, Australia

**Keywords:** Bortezomib, Carboplatin, Cisplatin, Copper transporter 1, Drug combination, Resistance, Oxaliplatin, Proteasomal degradation

## Abstract

**Background:**

Ovarian cancer remains an on-going challenge mainly due to the development of drug resistance and also because the cancer is likely to have metastasized at the time of diagnosis. Currently, chemotherapy based on platinum drugs such as cisplatin is the primary treatment for the disease. Copper transporter 1 is involved in the transport of cisplatin into the cell, but is also down-regulated by the drug. Bortezomib, a proteasome inhibitor, has been reported to block this platinum-induced down-regulation of CTR1, so that in the presence of bortezomib, the cellular uptake of platinum drugs may be increased. Increased platinum accumulation may result in increased platinum − DNA binding so that the platinum drug in combination with bortezomib may produce enhanced cell kill.

**Methods:**

In this study the efficacy of the sequential combinations of carboplatin, oxaliplatin and a *trans*-platinum compound coded as CH1 with BORT on the human ovarian A2780, A2780^cisR^, A2780^ZD0473R^ and SKOV-3 cancer cell lines was evaluated. The levels of cellular platinum accumulation and platinum-DNA binding were determined following the treatment with these combinations. In order to investigate the effect of the combinations of the formation of ROS, the total and oxidized glutathione levels were also determined.

**Results:**

Prevention of copper transporter 1 degradation by bortezomib is found to enhance the cellular accumulation of platinum, the level of Platinum − DNA binding and increases oxidative stress especially in the resistant cell lines.

**Conclusions:**

The results suggest that the prevention of CTR1 degradation by bortezomib may be playing a major role in increasing the cellular uptake of platinum drugs and platinum-DNA binding level. Furthermore, the generation of oxidative stress appears to be a major contributor to the enhanced cell kill.

## Background

Although platinum drugs cisplatin (CS), carboplatin (CB) and oxaliplatin (OX) are widely used alone and in combination with other drugs such as paclitaxel for treatment the of various cancers [1], their use has been limited due to dose-limiting toxicities, and intrinsic and/or acquired resistance leading to treatment failure [2]. Decreased cellular accumulation due to reduced drug intake and/or increased efflux [3-5], increased inactivation due to binding with glutathione or metallothionein, enhanced tolerance to platinum − DNA adducts and increased DNA repair are considered to be amongst the predominant mechanisms of resistance to platinum drugs [2,4].

In line with the idea that copper transporter 1 (CTR1) is a carrier for CS into the cell [6-10], it has been found that platinum accumulation in CTR1 knockout mice is markedly reduced [11] and its over-expression enhances the uptake [12,13]. Furthermore, the CS-resistant variant of ovarian A2780 cancer cell line has been found to have a reduced expression of hCTR1 mRNA. These results strongly suggest that efficacy due to platinum-based chemotherapy may be significantly improved through the modulation of CTR1 expression. It is important to note that like CTR1 that acts as the input carrier for Cu and Pt, P-type ATPases ATP7A and ATP7B are found to mediate both Cu and Pt efflux out of the cell [3,6,14].

Howell and co-workers have demonstrated that although CS is transported into the cell by CTR1, the drug triggers the proteasomal degradation of the carrier thereby limiting its own uptake [15], a process that can be prevented by pretreatment of cells with proteasomal inhibitors such as MG-132, lactacystin and bortezomib (BORT) [15,16]. An exception is the CTR1 expressed in human embryonic kidney cells that is not subject to CS-induced degradation, being stabilized as a multimeric complex [17]. Our recent studies have also confirmed that an increase in cell kill resulting from the combination of CS with BORT in ovarian tumour models is associated with an increase in cellular accumulation of CS and the level of Pt − DNA binding [18].

**Figure 1:**
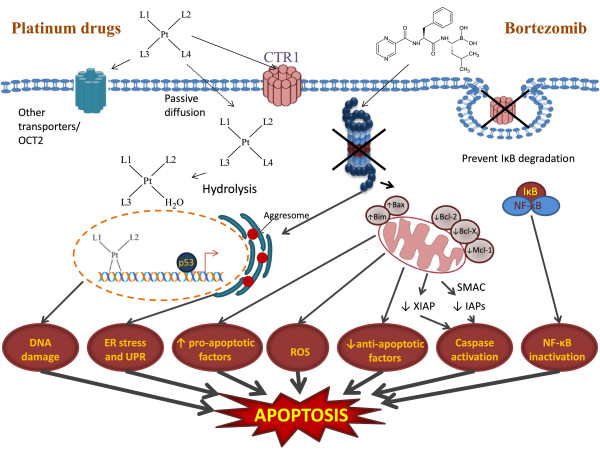
**Simplified representation of cellular events responsible for apoptosis due to combination of platinum drugs and bortezomib in which DNA damage, ER stress and increase in ROS due to platinum drugs and inactivation of antiapoptotic proteins such as NF-kB by bortezomib lead to apoptosis.** L1 and L2 denote the carrier ligands and L3 and L4 denote the leaving groups in CS, CB and OX. In CH1 which has a *trans*-geometry, L1 and L4 represent the carrier ligands and L2 and L3 represent the leaving groups.

Proteasome inhibition represents a unique approach to anticancer therapy as it targets the key regulator of intracellular protein degradation. In vitro studies have shown that the inhibition of the proteasome leads to the accumulation of inhibitor of κB (IκB) causing the down-regulation of the anti-apoptotic transcription factor NF-κB [19]. It also causes down regulation of other anti-apoptotic proteins such as MCL1, IAP and up regulation of pro-apoptotic proteins such as NOXA, p53, p27, BAX, BIM and SMAC [20]. Thus proteasome inhibition due to treatment with BORT can cause a shift in the balance between pro-apoptotic and anti-apoptotic factors towards apoptotic cell death, besides preventing the degradation of CTR1. BORT can also cause the production of reactive oxygen species (ROS) resulting into oxidative stress that further enhances the induction of apoptosis [21,22] (Figure 1).

**Figure 2:**
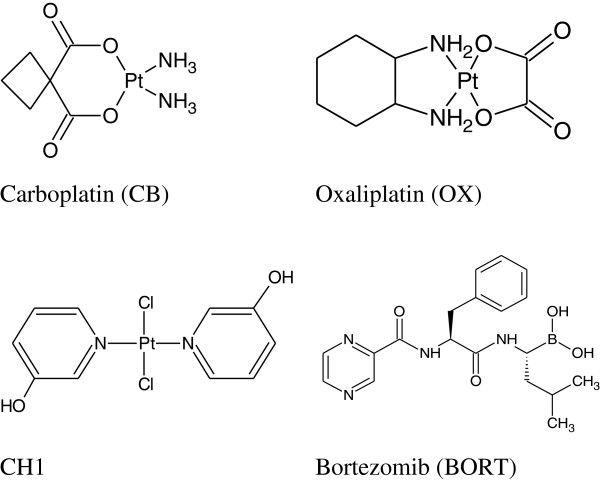
**Chemical structures of platinum compounds carboplatin (CB), oxaliplatin (OX), *trans***-**bis(3-hydroxypyridine)dichloroplatinum(II) (CH1), and that of proteasome inhibitor bortezomib (BORT), applied in combination to ovarian cancer cell lines.**

Human hCTR1 contains two methionine-rich motifs (hCTR1 Met) and two histidine-rich motifs (hCRT1 His) on its extracellular N-terminus that are thought to be essential for the function of the transporter. It has been shown that the interaction of CS, CB and OX with synthetic peptides corresponding to hCTR1Met motifs that contain three or more methionines result in the removal of the carrier ligands in the case of CS and CB [23] whereas OX is found to retain its DACH moiety (Figure 2). Recent studies by Wang et al. based on NMR spectroscopy and electrospray ionization mass spectrometry show that a maximum of two Pt atoms are bound to each monomer unit of hCTR1 for CB as well as for CS [24]. The binding to extracellular domain rather tight fit into any small pocket present in the carrier, leaves the door open for hCTR1 to serve as the influx carrier for larger platinum compounds such as OX, *trans*-planaramineplatinum(II) CH1 [25] and even polynuclear platinums such as BBR3464 and DH6Cl [26,27]. The present study aimed to determine the efficacy of sequential combinations of CB, OX and a *trans*-planaramineplatinum(II) coded as CH1 with BORT in ovarian tumour models [28].

## Methods

CB and OX were purchased from Sigma Aldrich, Sydney, Australia. BORT was purchased from LC Laboratories Woburn, MA, USA. The *Trans*-bis(3-hydroxypyridine)dichloroplatinum(II) coded as CH1 was synthesized in the host laboratory as described by Chowdhury et al. [25]. Foetal calf serum (FCS), RPMI-1640, 200 mM L-glutamine, and 5.6% sodium bicarbonate were purchased from Trace Biosciences Pty Ltd Australia. The DNA extraction kit JETQUICK Blood DNA Spin Kit/50 was obtained from Astral Scientific Pty Ltd, Sydney, Australia. GSH/GSSG-Glo™ assay kit was purchased from Promega, Sydney, Australia. All other chemicals were obtained from Sigma-Aldrich, Sydney, Australia. A2780, A2780^cisR^, A2780^ZD0473R^ and SKOV-3 ovarian cancer cell lines were gifts from Ms. Mei Zhang, Royal Prince Alfred Hospital, Sydney, Australia. Stock solutions (1 mM) of CB and OX were prepared in mQ water, that of CH1 prepared in 1:4 DMF to mQ water and that of BORT was made in ethanol. The solutions were sterilised by filtration.

### Cell culture

Human ovarian cancer cell lines A2780, A2780^cisR^, A2780^ZD0473R^ and SKOV-3 (Table 1) were seeded in 25 cm^2^ tissue culture flasks in a humidified atmosphere consisting of 5% CO_2_ and 95% air at 37°C. The cells in logarithmic growth phase were maintained in complete medium consisting of RPMI 1640, 10% heat inactivated FCS, 20 mM Hepes, 0.11% bicarbonate, and 2 mM glutamine without antibiotics. Each cell line was seeded in 10% FCS/RPMI 1640 culture medium at a density of 4000 and 5500 cells/well in flat-bottomed 96-well culture plate. The plate was then incubated for 24 h at 37°C in a humidified atmosphere to allow the cells to attach.

**Table 1:** Human ovarian cancer cell lines used in this study

**Cell line**	**Phenotype**
A2780	Untreated ovarian tumour
A2780^cisR^	CS resistant ovarian tumour
A2780^ZD0473R^	ZD0473 resistant ovarian tumour
SKOV3	Estrogen receptor positive ovarian tumour

### Single-drug treatment

Stock solutions of CB, OX, CH1 and BORT were subjected to serial dilutions to give final concentrations ranging from 0.0008 to 250 μM, made. The dilutions were made using 10% RMPI-1640 medium without serum and were added to equal volumes of cell culture in triplicate wells. Cells were treated with the drugs for 72 h in the incubator. Single drug treatments against each cell line were carried out to determine the (IC_50_) values i.e. drug concentrations required for 50% cell kill.

### Combination studies

Cells were treated with CB, OX, CH1 and BORT alone and in combinations (CB + BORT, OX + BORT and CH1 + BORT) at three different concentration. Three modes of administration: 0/0 h, 0/2 h and 2/0 h were used, where 0/0 h indicates that both the compounds were added simultaneously, 0/2 h means that the platinum drug was added first followed by BORT 2 h later and 2/0 h means that the platinum drug was added 2 h after the addition of BORT. The period of drug treatment was 72 h counted from the time of addition ofthe first compound. Cell growth inhibition was determined using the MTT reduction assay. Combination index values (CIs) were used as measures of synergism, additiveness or antagonism calculated using the program CalcuSyn [29-31]. The CI for binary combinations of drugs was calculated according to the equation:

$$\text{CI}=\frac{D_{1}}{D_{1x}}+\frac{D_{2}}{D_{2x}}$$

Where D_1_ and D_2_ respectively represent mean doses of compounds 1 and 2 in combination required to cause x% inhibition, whereas D_1×_ and D_2×_ represent the doses of compounds 1 and 2 respectively required to cause x% inhibition when present alone. D_x_ is calculated from the following median effect equation, where D_x_ denotes the dose of drug, D_m_ is the median-effect dose, f_a_ is the fraction of cells affected so that f_u_ = 1-f_a_ and m is the exponent defining the shape of the dose effect curve. CI values of <1, =1 and >1 indicate synergism, additivity and antagonism in combined drug action, respectively.

$$D_{\times }=D_{m}\;X\;{\left[f_{a}/\left({1‒f}_{a}\right)\right]}^{1/m}$$

### Platinum cellular accumulation and platinum − DNA binding studies

The cellular accumulation of platinum and platinum − DNA binding levels from the 0/0 h and 0/2 h combinations of CB and OX with BORT in A2780 and A2780^cisR^ cell lines were determined. Combinations of the drugs at their IC_50_ values were added to culture plates containing exponentially growing A2780 and A2780^cisR^ cells in 10 mL 10% FCS/RPMI 1640 culture medium with cell density of 5 × 10^6^ cells mL^–1^ and incubated for 24 h. The cells were scraped off the culture plates and transferred to 10 mL centrifuge tubes and spun at 3500 rpm for 2 min at 4°C. The cells were washed thrice with ice-cold phosphate-buffered saline (PBS) and the pellets were stored at −20°C until assayed. A minimum of three independent experiments were performed.

### Cellular accumulation

Following drug treatments and collection, the cell pellets were resuspended in 0.5 mL 1% triton-X and sonicated for 30 min on ice. The total intracellular content of platinum was determined by graphite furnace atomic absorption spectrophotometry.

### Platinum–DNA binding

The DNA isolated from cell pellets using JETQUICK Blood DNA Spin Kit/50 Astral Scientific Pty Ltd were analysed for it platinum bound content by graphite furnace AAS. A_260_/A_280_ nm ratios were between 1.75 and 1.8 for all samples indicating high purity of the DNA.

### Cellular glutathione

As a measure of the redox state of the cells, the levels of total glutathione (GSH and GSSG) as well as oxidised glutathione (GSSG) in A2780 and A2780^cisR^ cell lines were determined for the 0/0 h and 0/2 h sequenced combinations of CB and OX with BORT. Drugs made in 10% RMPI-1640 serum free medium were added to equal volumes of cell culture wells of a white wall clear bottom 94 well plate containing exponentially growing A2780 and A2780^cisR^ cells (cell density = 12 × 10^3^ cells/well). Cells were left to incubate for 24 h. The media was aspirated out of the treatment wells with minimal disturbance of the cell pellets and cells were washed with 200 μL of PBS following which the levels of glutathione were determined using the GSH/GSSG-Glo™ Assay kit (Promega). The plate was read in a LUMIstar Omega luminometer (BMG LABTECH, USA).

## Results

### Cytotoxicity

**Figure 3:**
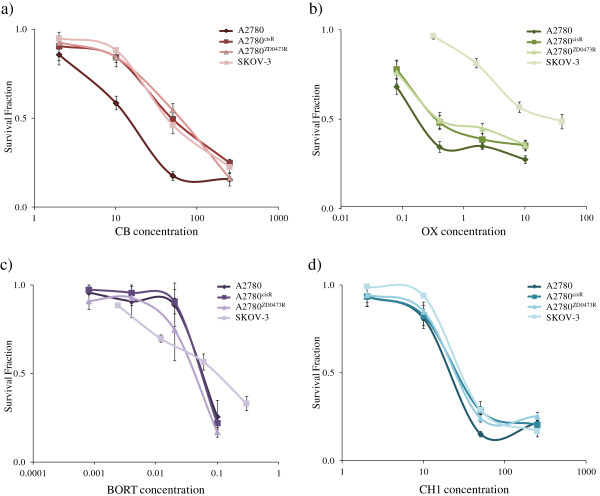
**Inhibition of cell growth as a function of increasing concentrations of a**) **CB**, **b**) **OX c**) **CH1 and d**) **BORT as applied to the ovarian cancer cell lines A2780**, **A2780**^**cisR**^, **A2780**^**ZD0473R **^**and SKOV**-**3.** Cell survival fractions following treatment with increasing concentrations of drugs for 72 h were determined using MTT assay and spectrophotometric analysis. Error bars represent the standard deviation.

Figure 3 shows the cell survival fraction versus concentration plots for CB, OX, CH1 and BORT as applied to the human ovarian cancer cell line A2780, A2780^cisR^, A2780^ZD0473R^ and SKOV-3. The parent A2780 cell line was most sensitive to the drugs. A2780^ZD0473R^ was the most resistant to CB whereas SKOV-3 was the most resistant to CH1, OX and BORT. The IC_50_ values of the compounds against the cell lines are presented in Table 2. The IC_50_ values for CB and OX are found to be greater in the resistant A2780^cisR^, A2780^ZD0473R^ and SKOV-3 cell lines with OX having very high value in SKOV-3. The activity of CH1 on the other hand is found to be comparable against all the cell lines so that it has the lowest resistance factors as compared to CB and OX. The IC_50_ values for BORT against A2780^cisR^ and A2780^ZD0473R^ are found to be nearly the same as that against the parent A2780 cell line and slightly greater against SKOV-3 cell line. Furthermore, BORT is found to be significantly more active than CB, OX and CH1 against all four human ovarian cancer cell lines. This work does not require any ethical approval as it does not involve animals and humans.

**Table 2:** **Summary of the IC**_**50 **_**values** (μ**M**) **and Resistance Factors** (**RF**) **for CS**, **CB**, **OX**, **CH1 and BORT as applied to the ovarian cancer cell lines A2780**, **A2780**^**cisR**^, **A2780**^**ZD0473R **^**and SKOV**-**3**

	**A2780**	**A2780**^**cisR**^	**RF**	**A2780**^**ZD0473R**^	**RF**	**SKOV**-**3**	**RF**
CS	0.5 ± 0.03	8.2 ± 0.6	17.7	6.0 ± 0.5	13.0	10.2 ± 0.5	22.2
CB	14.0 ± 1.4	48.9 ± 3.9	3.5	64.6 ± 3.2	4.6	43.4 ± 3.9	3.1
OX	0.2 ± 0.01	0.4 ± 0.02	1.9	0.4 ± 0.03	2.1	43.6 ± 3.0	229.4
CH1	21.3 ± 2.0	26.6 ± 1.9	1.2	25.4 ± 1.7	1.2	29.8 ± 1.8	1.4
BORT	0.05 ± 0.01	0.05 ± 0.01	1.0	0.04 ± 0.01	0.8	0.09 ± 0.01	1.8

*RF* is defined as the ratio of the *IC*_50_ value in the resistant cell line over that in the responsive parent cell line.

### Combination studies

**Figure 4:**
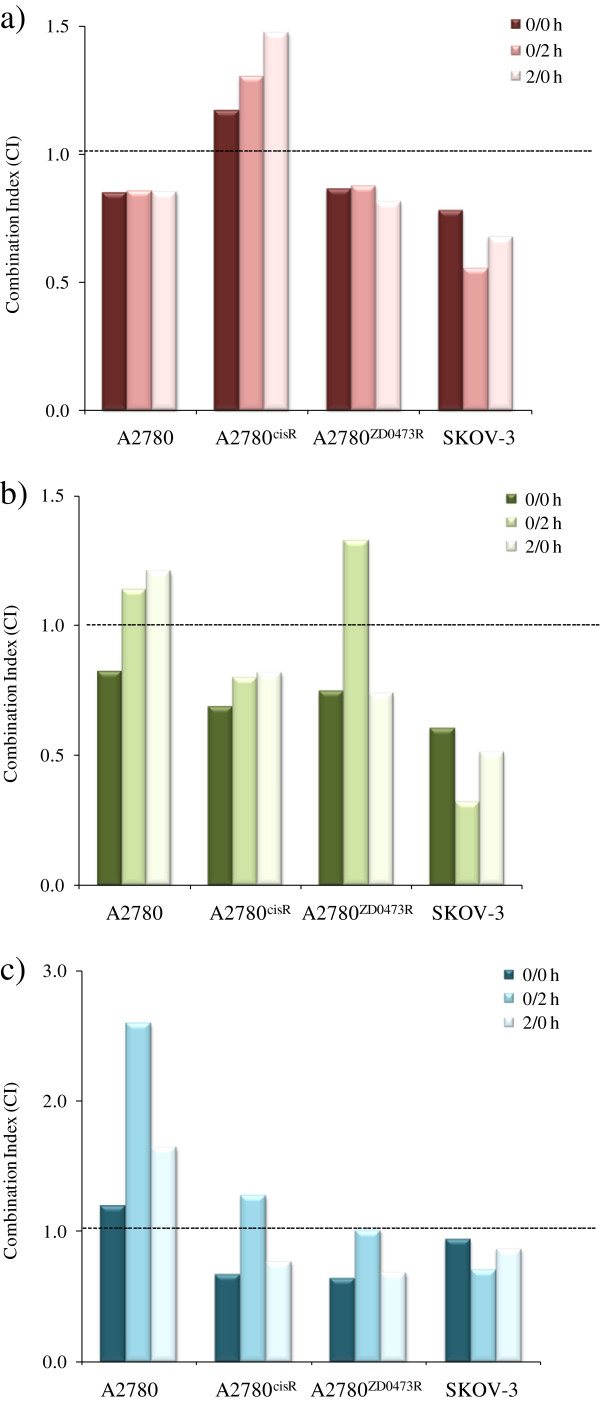
**Combination Index (CI) values applying to the sequenced combinations of a) CB, b) OX and c) CH1 with BORT administered to the ovarian A2780, A2780**^**cisR**^**, A2780**^**ZD0473R **^**and SKOV-3 cancer cell lines.** CI values were calculated following 72 h treatments with the drugs at their equipotent ratios. CI values of <1, =1 and >1 indicate respectively synergism, additivity and antagonism in combined drug action.

Figure 4 a, b, c and d show respectively the combination index values [32] for the combinations of CB, OX and CH1 with BORT in: A2780, A2780^cisR^, A2780^ZD0473R^ and SKOV-3 cell lines. Combinations of CB with BORT were found to be synergistic in A2780, A2780^ZD0473R^ and SKOV-3 cell lines irrespective of the sequence of administration with the greatest cell kill resulting from the 0/2 h sequence. In the CS resistant cell line A2780^cisR^, all combinations of CB and BORT produced pronounced cell death. The SKOV-3 cell line also responded well to the combination of OX with BORT with greatest synergism being observed with the 0/2 h sequence of administration. The bolus administration of OX and BORT resulted in synergism in A2780 whereas 0/2 h and 2/0 h sequences of administration were slightly antagonistic. On the other hand, the bolus and 2/0 h sequence of combinations of OX with BORT caused synergism in A2780^ZD0473R^ cell line while 0/2 h sequence of administration was found to be antagonistic. The combinations of the *trans*-platinum CH1 with BORT were also found to be synergistic in A2780^ZD0473R^, SKOV-3 and A2780^cisR^ cells except for the 0/2 h sequence of administration in A2780^cisR^. The 0/2 h sequence of administration was also antagonistic in the parent A2780 cell line.

### Platinum accumulation

**Figure 5:**
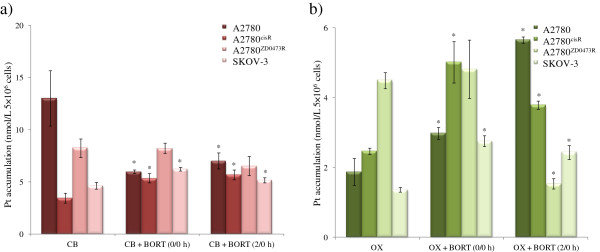
**Cellular platinum accumulation in ovarian cancer A2780 and A2780**^**cisR**^**, A2780**^**ZD0473R **^**and SKOV-3 cell lines as applied to single treatments of CB and OX and their selected combinations with BORT.** Cells were treated with the drugs for 24 h followed by collection, lysis and finally the detection of Pt was using AAS. Data were statistically analyzed using the paired Student’s t test: * *p* <0.05 indicates significant difference from control. Error bars represent the standard deviation.

To determine whether the presence of BORT led to an enhancement in the uptake of CB and OX, the level of intracellular platinum in A2780 and A2780^cisR^, A2780^ZD0473R^ and SKOV-3 cell lines were determined after 24 h treatment for each drug combination (Figure 5). It was found that the intracellular accumulation of platinum from CB alone was greater in the parent A2780 cell line than in the resistant A2780^cisR^ cell line whereas the converse was true from OX. Also the presence of BORT was found to increase the accumulation of CB in the resistant A2780^cisR^ cell line (but not in the parent A2780 cell line) for both 0/0 h and 2/0 h sequences of administration (more so for the 2/0 h sequence of administration). The presence of BORT was also found to increase cellular accumulation of CB in SKOV-3 cell line but more so for the 0/0 h sequence of administration than 2/0 h sequence of administration. The presence of BORT was not found to have significant on cellular of CB in A2780^ZD047R^ cell line. As applied to the parent A2780 cell line, on face value, served to decrease rather than increase the cellular accumulation of CB although uncertainty remains because of large error. A further point to note that increase in accumulation of CB in A2780^cisR^ cell line, did not result in any increase in the cell kill. The cellular accumulation of OX was found to be highest in the resistant A2780^cisR^ cell line but lower than that of CB in all the four cell lines. As applied to the combination of OX with BORT, 0/0 h sequence of administration resulted in highest platinum accumulation in the resistant A2780^cisR^ cell line whereas 2/0 h sequence of administration resulted in highest platinum accumulation in the parent A2780 cell line.

### Platinum − DNA binding

**Figure 6:**
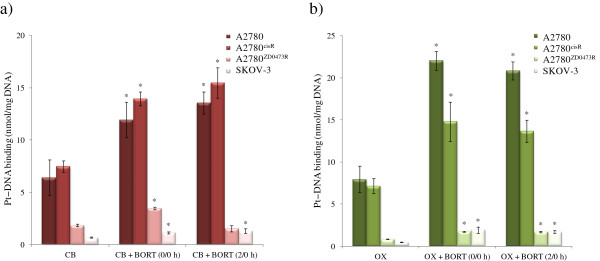
**Platinum − DNA binding levels in the ovarian A2780, A2780**^**cisR**^**, A2780**^**ZD0473R **^**and SKOV-3 cancer cell lines due to treatment with CB and OX and their combinations with BORT.** Cells were treated with the drugs for 24 h followed by collection, DNA extraction and finally the detection of Pt using AAS. Data were statistically analyzed using the paired Student’s *t* test: * *p* <0.05 indicates significant difference from control. Error bars represent the standard deviation.

As the action of platinum drugs is associated with their binding with DNA, platinum − DNA binding levels in A2780 and A2780^cisR^, A2780^ZD0473R^ and SKOV-3 cell lines were determined for the 0/0 h and 2/0 h combinations of CB and OX with BORT. Figure 6 shows the platinum − DNA binding levels in A2780 and A2780^cisR^, A2780^ZD0473R^ and SKOV-3 cell lines resulting from CB and OX alone and from the 0/0 h and 2/0 h combinations of CB and OX with BORT. Platinum − DNA binding level from CB alone was found to be highest in the resistant A2780^cisR^ cell line and from OX alone it was highest in the parent A2780 cell line.

Platinum − DNA binding levels from the combinations of CB and OX with BORT were found to be greater than those from CB and OX alone in both the parent A2780 and the resistant A2780^cisR^ cell lines. The levels in A2780^ZD047R^ and SKOV-3 cell lines were found to be much lower from the drugs alone as well as their combinations with BORT. A more careful analysis shows that 0/0 h combination of CB with BORT resulted in a significant increase in platinum − DNA binding level in both A2780^ZD047R^ and SKOV-3 cell lines. As applied to combination of OX with BORT, both the sequences of administration resulted in increase in platinum − DNA binding in all the four cell lines A2780, A2780^cisR^, A2780^ZD047R^ and SKOV-3.

### Cellular glutathione

**Figure 7:**
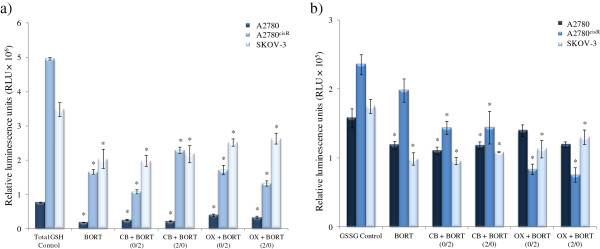
**Levels of reduced (GSH) and oxidized (GSSG) forms of cellular glutathione in relative luminescence units (RLU** **×** **10**^**5**^**) before and after treatments with BORT alone and its combination with CB and OX in the ovarian cancer A2780**, **A2780**^**cisR **^**and SKOV-3 cell lines.** Cells were treated for 24 h and glutathione content was determined using GSH/GSSG-Glo Assay kit. Data were statistically analyzed using the paired Student’s *t* test: * *p* <0.05 indicates significant difference from control. Error bars represent the standard deviation.

As both platinum drugs and BORT are able to induce oxidative stress in the cells that may also lead to apoptosis, the effect of the drug combination on cellular glutathione levels was investigated. Figures 7 a and b show the levels of total glutathione (GSH and GSSG) and oxidized glutathione (GSSG) (in relative luminescence units) in A2780, A2780^cisR^ and SKOV-3 cell lines after treatment of cells with combinations of BORT with CB and OX, administered using 0/0 h and 0/2 h sequences. The level of glutathione in A2780^ZD0473R^ cell line was not determined to minimise cost. Table 3 gives the ratios of GSH/GSSG before and after treatments with BORT and its combinations with CB and OX.

**Table 3:** **Ratios of GSH/GSSG before and after treatments with BORT and its combinations with CB and OX in A2780, A2780**^**cisR **^**and SKOV-3 ovarian cancer cell lines**

	**A2780**	**A2780**^**cisR**^	**SKOV**-**3**
Control	241.32	40.2	38.00
BORT	31.3	14.7	42.56
CB + BORT (0/2 h)	78.35	13.1	38.53
CB + BORT (2/0 h)	50.27	13.8	35.70
OX + BORT (0/2 h)	71.77	39.25	38.16
OX + BORT (2/0 h)	69.97	33.31	43.74

Both total and oxidised glutathione levels were found to be highest in the CS-resistant A2780^cisR^ cell line and lowest in the parent A2780 cell line. Treatment with BORT alone also caused a significant decrease in GSH level in A2780, A2780^cisR^ and SKOV-3 cell lines, thus indicating the heightening of oxidative stress.

It can be seen that treatments with BORT and its combinations with CB and OX have served to decrease values for GSH/GSSG ratio relative to the control – more so in the resistant A2780^cisR^ and SKOV-3 cell lines than in the parent A2780 cell line.

## Discussion

In this study, efficacy of sequenced combinations of CB, OX and CH1 with proteasome inhibitor BORT in human ovarian A2780, A2780^cisR^, A2780^ZD0473R^ and SKOV-3 cell lines was determined, as differences in mechanism of action suggest that the drugs might act synergistically in combination. Besides being a highly potent anticancer drug on its own right, BORT can also enhance the activity of platinum drugs by counteracting platinum-induced loss of CTR1 expression [16]. Amongst the three platinum compounds, OX was most active and CH1 was least active against the parent cell line A2780. CB was the least active compound against the resistant A2780^cisR^ and A2780^ZD0473R^ cell lines. Although both CB and CS form intrastrand bifunctional adducts with DNA (that are followed by protein recognition and cascade of events leading to programmed cell death), the two compounds differ in their leaving groups (chloride in CS and cyclobutanedicarboxylate (CBDCA) ligand in CB) and consequently in their reactivity. The ligand exchange reactions with carboxylate groups are much slower than those with the chloride ligand so that CB is significantly more stable than CS [33]. NMR study has revealed that the activation of CB requires the opening of the CBDCA ring and that the rate of ring opening is strongly dependent on the availability of nucleophiles, especially sulfur-containing ones [34]. The lower reactivity of CB relative to CS, serves to lengthen the time required for its aquation and subsequent formation of adducts with DNA [35]. Consequently, CB is 4- to 10-fold less potent than CS in various tumour cell types as evident from differences in IC_50_ values of the two compounds [33]. However, tumour cell lines resistant to CS have been found to be cross-resistant to CB, a fact that has been attributed to the formation of identical adducts with DNA [36].

Much greater activity of OX than CS against A2780, A2780^cisR^ and A2780^ZD0473R^ cell lines may be due to differences in their structures in terms of both the leaving groups (oxalate in OX as against Cl^-^ in CS) and the carrier ligands (*trans*-*R*,*R*-diaminocyclohexane abbreviated as DACH present in OX as against NH_3_ in CS). Although OX, having a *cis*-geometry like CS, can form intrastrand 1,2-(GpG) adducts with DNA, several conformational differences exist in the intrastrand 1,2-(GpG) adducts formed by CS and OX [37,38]. These conformational differences may be responsible for differences in protein recognition and cellular processing, thus providing an explanation (at least in part) as to why OX–DNA adducts are not recognized by mismatch repair proteins so that OX has a higher activity than CS in CS-resistant tumours [39]. Much lower activity of OX against SKOV-3 cell line may be due to p53-null status of the cells. Mutations of p53 in cancer cells invariably abolish its activity, due to the pro-apoptotic role played by p53 in tumour suppression [40]. In a panel of colon cancer cell lines, sensitivity to OX was found to be characteristic of p53 wild-type cells whereas p53 mutated-cells exhibited a marked increase in resistance to OX [41]. Further work needs to be carried out to fully elucidate the mechanisms of resistance in SKOV-3 against OX. Although the *trans*-platinum compound CH1 has a relatively lower activity than *cis*-platinums against all four ovarian cancer cell lines, it has lower resistance factors, indicating that at the level of its activity CH1 has been better able to overcome mechanisms of resistance operating in A2780^cisR^, A2780^ZD0473R^ and SKOV-3 cell lines.

BORT has shown remarkably high activity against all the four human ovarian cancer cell lines. Inhibition of proteasome leads to the up-regulation of pro-apoptotic proteins such as inhibitor of κB (IκB), p53 and NOXA and down-regulation of anti-apoptotic proteins such as MCL1, IAP [19,20], thus enabling BORT to induce apoptosis independent of platinum action (in terms of binding to the DNA).

Combinations of CB with BORT were found to be synergistic in A2780, A2780^ZD0473R^ and SKOV-3 cell lines irrespective of sequence of administration but antagonistic in A2780^cisR^ cell line. The synergism in activity from 0/0 h and 2/0 h combinations of CS with BORT in A2780 and A2780^cisR^ cell lines is in line with the increased cellular accumulation of platinum and increased level of Pt − DNA binding. In a phase I clinical trial, the combination of CB with BORT has shown promising results [42]. BORT decreased CB-induced NF-κB activity with 47% overall response rates, two complete responses (CR) and five partial responses, including one CR in a patient with platinum-resistant disease [42]. In the present study, combinations of CB with BORT were not found to cause any enhancement of cell kill in the CS-resistant cell line (in actual fact the combinations were antagonistic in action), although both the cellular platinum accumulation of platinum and the level of Pt − DNA binding were elevated in A2780 and A2780^cisR^ cell lines. It is likely that much higher activity of BORT against both A2780 and A2780^cisR^ cell lines but much lower activity of CB against A2780^cisR^ cell line than the parent A2780 cell line, has served to dampen the effect of increase in Pt − DNA level that results from the protective role played by BORT against CTR1 degradation. As applied to the combinations of BORT and OX also, both cellular accumulation of platinum and the level of Pt − DNA binding were found to be greater than those from OX alone in both the cell lines. The increase in platinum uptake and the level of Pt − DNA binding from OX in the presence of BORT suggests that CTR1 can also serve as a carrier for the much larger molecule OX. In the case of the much larger molecule CH1, it appears that this compound also acts synergistically in combination with BORT in A2780^cisR^, A2780^ZD0473R^ and SKOV-3 cell lines (although not so in the parent A2780 cell line) suggesting that BORT may be acting as a carrier for OX and CH1 as well. This is not unexpected as the association between CTR1 and platinum drugs does not involve tight fit into a small pocket. Surprisingly, the SKOV-3 cell line that showed marked resistance to OX was most responsive to the combination of OX with BORT, indicating that the presence of BORT had served to greatly sensitize the SKOV-3 cells to cell kill due to OX. The cellular accumulation of platinum from combinations of OX with BORT are found to be higher (than those from OX alone) in all the four A2780, A2780^cisR^, A2780^ZD0473R^ and SKOV-3 cell lines as applied to the 0/0 h sequence of administration and in A2780, A2780^cisR^ and SKOV-3 cell lines as applied to 2/0 h sequence of administration and the levels of platinum − DNA binding are greater in A2780, A2780^cisR^, A2780^ZD0473R^ and SKOV-3 cell lines (more so in A2780 and A2780^cisR^ cell lines ) for both 0/0 h and 2/0 h sequences of administration. The results can be seen to be in line with synergistic nature of the combinations. Finally, the results indicate the combinations of CB, OX and CH1 with BORT generally serve to enhance cell kill especially in the resistant cell lines.

As BORT and platinum drugs are known to cause oxidative stress in cancer cells, the level of cellular glutathione was determined for the combinations of BORT with CB and OX. It was found that the treatment of A2780, A2780^cisR^ and SKOV-3 ovarian cancer cells with BORT alone and its combinations with CB and OX significantly reduced the total glutathione (GSH and GSSG) levels in all the three cell lines – more so from BORT alone than from the combinations. The results indicate that the proteasome inhibitor BORT induces a greater oxidative stress in cancer cells than platinum drugs CB and OX although all the three compounds − BORT, CB and OX − can induce oxidative stress in the cells. The change was found to be more significant for the reduced form GSH than the oxidized form GSSG so that treatments with BORT and its combinations with CB and OX have served to decrease the values for GSH/GSSG ratio (relative to the control) and more so in the resistant A2780^cisR^ and SKOV-3 cell lines than in the parent A2780 cell line. The results indicate that treatment of the cells with BORT and its combinations with CB and OX have served to heighten oxidative stress in the cells.

Contrary to the common observation that the oxidised form of glutathione (GSSG) is elevated following oxidative stress, in the present study it was found that the level of GSSG decreased (although only slightly) after the drug treatments. It is important to note that GSSG may either recycle to GSH or exit from the cells, leading to the overall depletion of cellular glutathione content [43]. The fact that both reduced and oxidised forms of glutathione decreased following drug treatment means that it is more likely that the extrusion of glutathione has occurred, possibly through the multidrug resistance-associated protein (MRP) [44].

In summary, the key points in regard to the combinations of platinum drugs and BORT administered to ovarian tumour models are: i. Proteasomal degradation of CTR1 induced by CS and possibly by other platinum drugs so that CS serves to decrease its own uptake, ii. BORT plays a protective role against CS-induced proteasomal degradation of CTR1 so that in presence of BORT cellular accumulation of platinum and the level of platinum-DNA binding is enhanced, iii. Protein recognition of platinum − DNA lesions may initiate a cascade of events leading to apoptosis or repair of the DNA lesions causing drug resistance, iv. BORT causes death of cancer cells through proteasome inhibition, v. both platinum drugs and BORT cause apoptosis through oxidative stress, and vi. the much greater activity of BORT as compared to platinum drugs, especially in the resistant tumour models, masks the effects of CTR1-prtoection. Figure 1 gives a pictorial representation of key events associated with the combination of platinum drugs with BORT administered to ovarian cancer cells.

## Conclusion

The increase in cellular accumulation of platinum and the level of Pt − DNA binding associated with combination of BORT with CB and OX in ovarian tumour models indicate that BORT may serve to protect CTR1 from CS-induced proteasomal degradation. However, the effect on the cell kill appears to be less significant due to much lower activity of platinum drugs as compared to BORT.

## Abbreviations

BORT: Bortezomib; CB: Carboplatin; CBDCA: Cyclobutanedicarboxylate; CH1: *Trans*-bis(3-hydroxypyridine)dichloroplatinum(II); CI: Combination index; CS: Cisplatin; CTR1: Copper transporter 1; Cu: Copper; DACH: Diaminocyclohexane; FCS: Foetal calf serum; GSH: Glutathione; GSSG: Oxidised glutathione; His: Histidine; IκB: Inhibitor of Kappa B; Met: Methionine; MRP: Multidrug resistance-associated protein; MTT: 3-(4,5-Dimethylthiazol-2-yl)-2,5-diphenyltetrazolium bromide; NF-κB: Nuclear factor Kappa B; OX: Oxaliplatin; PBS: Phosphate buffered saline; Pt: Platinum; ROS: Reactive oxygen species.

## Competing interests

The authors declare that they have no competing interests.

## Authors’ contributions

ZA developed the methodology, acquired and interpreted data, and drafted the manuscript. PB, CC and JQY aided in study design. FH conceived and designed the study, developed the methodology, interpreted data, edited the manuscript, and oversaw the study. All authors have read and approved the final manuscript.
